# Comparative Evaluation of Natural Mouthrinses and Chlorhexidine in Dental Plaque Management: A Pilot Randomized Clinical Trial

**DOI:** 10.3390/healthcare13101181

**Published:** 2025-05-19

**Authors:** Ioana Elena Lile, Tareq Hajaj, Ioana Veja, Tiberiu Hosszu, Ligia Luminița Vaida, Liana Todor, Otilia Stana, Ramona-Amina Popovici, Diana Marian

**Affiliations:** 1 Department of Dentistry, Faculty of Dentistry, “Vasile Goldiș” Western University of Arad, 94-96 Revolutiei Blvd., 310025 Arad, Romania; lile.ioana@uvvg.ro (I.E.L.); hosszu.tiberiu@uvvg.ro (T.H.); marian.diana@uvvg.ro (D.M.); 2 Department of Propaedeutics and Dental Materials, Faculty of Dentistry, Victor Babes University of Medicine and Pharmacy, 2 Eftimie Murgu Sq, 300041 Timisoara, Romania; tareq.hajaj@umft.ro; 3 Research Center in Dental Medicine Using Conventional and Alternative Technologies, Faculty of Dental Medicine, Victor Babes University of Medicine and Pharmacy of Timisoara, 9 Revolutiei 1989 Ave, 300070 Timisoara, Romania; 4 Department of Dentistry, Faculty of Medicine and Pharmacy, University of Oradea, 1 Universitatii Street, 410087 Oradea, Romania; ligia_vaida@uoradea.ro (L.L.V.); liana.todor@uoradea.ro (L.T.); 5 Management and Communication in Dental Medicine Department I, Faculty of Dental Medicine, Victor Babes University of Medicine and Pharmacy, Piața Eftimie Murgu 2, 300041 Timisoara, Romania; ramona.popovici@umft.ro

**Keywords:** dental plaque, oral health, propolis, green tea, chlorhexidine, oral hygiene

## Abstract

**Aim:** This study evaluated the efficacy of mouthrinses containing natural compounds—specifically, propolis and green tea extracts—in reducing bacterial dental plaque compared to a placebo and a 0.2% chlorhexidine rinse. We hypothesized that these natural compounds would significantly reduce plaque accumulation, with efficacy comparable to chlorhexidine. **Objective:** The objective was to evaluate the short-term efficacy of two natural mouthrinses—10% propolis and 5% green tea—compared to a placebo and 0.2% chlorhexidine in reducing dental plaque. **Trial Design:** The trial design was a randomized, placebo-controlled, parallel-group clinical trial with a 1:1:1:1 allocation ratio. **Materials and Methods:** In a single-blind, randomized, controlled trial, 60 healthy adult volunteers received a professional mechanical plaque removal (PMPR) and were then randomized into four groups (*n* = 15 each): a propolis mouthwash, a green tea mouthwash, a 0.2% chlorhexidine mouthwash (positive control), and a placebo rinse. The participants rinsed twice daily for four weeks in addition to standard tooth brushing. The plaque levels were assessed using the Silness–Löe plaque index at baseline and after four weeks. The data were analyzed using ANOVA and post hoc tests (α = 0.05). Ethical approval and informed consent were obtained. **Results:** All groups had similar baseline plaque scores (≈2.5). After four weeks, the propolis and green tea groups showed significant reductions in plaque (mean indices of 1.02 and 1.12, respectively) compared to the placebo group (mean index = 2.01, *p* < 0.001). The chlorhexidine group achieved a mean plaque index of 0.90. The propolis rinse showed no significant difference from chlorhexidine (*p* = 0.40), indicating comparable efficacy. The green tea rinse had a slightly higher plaque index than chlorhexidine (*p* = 0.03). No significant adverse effects were reported. **Conclusions:** Mouthwashes containing 10% propolis or 5% green tea significantly reduced dental plaque, with propolis demonstrating efficacy comparable to 0.2% chlorhexidine.

## 1. Introduction

Bacterial dental plaque forms rapidly on tooth surfaces and serves as a primary etiological factor for both dental caries and periodontal disease [1]. Effective plaque control is, therefore, essential for maintaining oral health, as it reduces the risk of both carious and inflammatory conditions [2]. Among the available chemical agents, chlorhexidine (CHX) is widely regarded as the gold standard for chemical plaque control due to its broad-spectrum antimicrobial action and high substantivity [3]. It binds to oral tissues and tooth surfaces, offering prolonged antibacterial effects against key pathogens, such as Streptococcus mutans and Porphyromonas gingivalis [4]. However, its long-term use is frequently associated with undesirable side effects—including tooth staining, altered taste perception, and mucosal irritation—which limit patient compliance and long-term applicability [5,6].

These limitations have spurred increasing interest in natural, plant-based alternatives with antiplaque and anti-inflammatory properties but fewer adverse effects. Among the most promising are propolis and green tea (Camellia sinensis) extracts. Propolis, a resinous substance produced by honeybees, contains over 300 bioactive compounds, notably flavonoids and phenolic acids, known for their potent antimicrobial, anti-inflammatory, and anti-biofilm effects [7,8]. In dentistry, propolis has been shown to inhibit plaque accumulation, reduce gingival inflammation, and prevent bacterial adhesion [9]. A recent randomized controlled trial (2024) demonstrated that a 0.2% propolis mouthrinse significantly reduced plaque index scores over a three-week period, with outcomes comparable to those of a 0.2% CHX rinse [10].

Green tea is another plant-derived agent with well-documented oral health benefits. It is rich in catechins, particularly epigallocatechin-3-gallate (EGCG), which exert antibacterial and anti-biofilm activity by disrupting bacterial adhesion, acid production, and glucosyltransferase activity [11,12]. Clinical studies report that green tea mouthrinses can reduce dental plaque and gingival inflammation and, in some cases, demonstrate efficacy comparable to chlorhexidine [13,14]. Moreover, green tea rinses are generally well tolerated, with minimal reports of adverse effects, such as staining or taste alteration, making them suitable for long-term use [15].

Despite these promising findings, many existing studies on natural antiplaque agents are limited by small sample sizes, methodological heterogeneity, or short follow-up durations. Therefore, further well-designed clinical trials are warranted to determine the comparative efficacy of natural agents versus conventional treatments, like CHX [16,17].

In this context, the current pilot randomized controlled trial was designed to compare the short-term plaque-reducing efficacy of two alcohol-free mouthrinses—containing either 10% propolis or 5% green tea extract—with a 0.2% chlorhexidine rinse (positive control) and a placebo. We hypothesized that these natural compound-based rinses would significantly reduce plaque accumulation and demonstrate efficacy comparable to that of chlorhexidine.

## 2. Materials and Methods

This study was designed as a single-blind, randomized controlled trial with four parallel groups. Sixty (60) adult volunteers, aged 18–50, were recruited from the university clinic. All participants were in good general health and had evidence of dental plaque accumulation (moderate plaque index scores) but no advanced periodontal disease. Baseline evaluations included medical and dental history and oral examination. This study was conducted in compliance with ethical standards for human research. This study was registered with the ISRCTN Registry (ISRCTN16734449), and ethical approval was obtained from the Institutional Ethics Committee of Vasile Goldiș Western University of Arad (Approval No. 01/10.01.2025). All participants provided written informed consent after receiving a full explanation of the study procedures, benefits, and potential risks. Participants were informed of their right to withdraw from the study at any time. Throughout the study, the principles of the Declaration of Helsinki were adhered to, ensuring respect for persons, beneficence, and confidentiality of participant information.

### 2.1. Inclusion Criteria

- Age 18–50 years, systemically healthy.
- Minimum of 20 natural teeth present.
- Moderate plaque accumulation (baseline plaque index score between 1.5 and 3.0).
- No professional dental cleaning within the past 3 months.

### 2.2. Exclusion Criteria

- Any systemic condition requiring antibiotic prophylaxis.
- Use of antibiotics or antiseptic mouthrinses in the last 4 weeks.
- Ongoing dental treatment or recent periodontal therapy.
- Known allergy to any product ingredients (propolis, tea, or chlorhexidine).
- Pregnant or lactating women.

**Figure 1:**
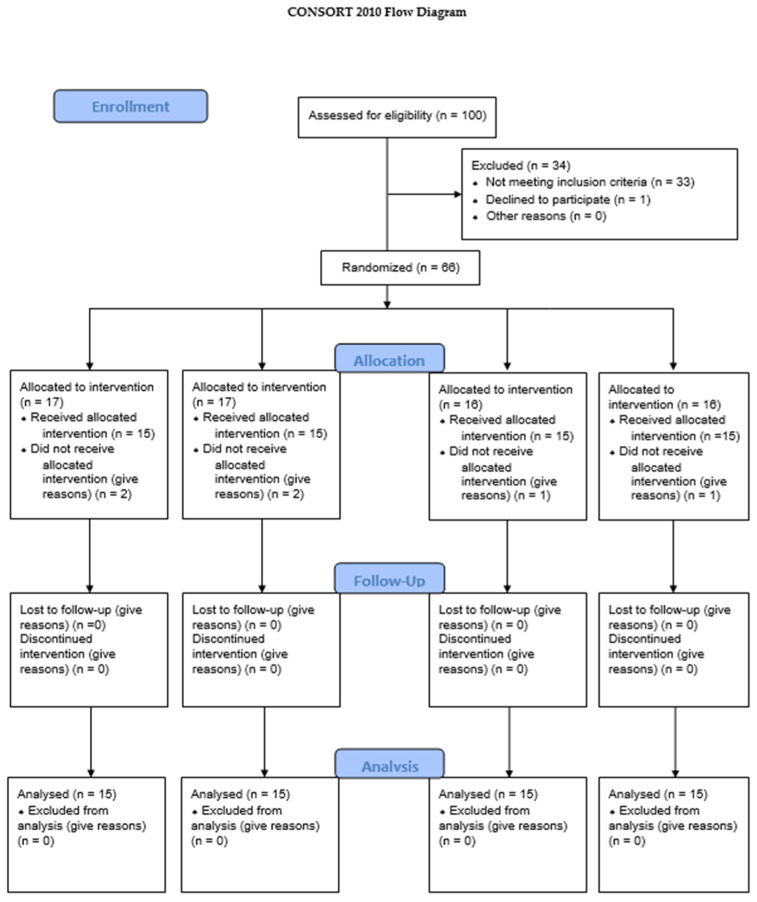
Consort 2010 flow diagram.

Participants who met the criteria and provided written informed consent were enrolled. The sample size (15 subjects per group, total n = 60) was determined based on a power analysis to detect a clinically significant difference in plaque index between groups (power 80%, α = 0.05). All eligible participants received a professional mechanical plaque removal (PMPR) at baseline to ensure a uniform starting point with minimal plaque. This “zero baseline” prophylaxis allowed for the measurement of new plaque accumulation over the study period (Figure 1).

Before randomization and the initiation of the rinsing protocol, all participants underwent a professional mechanical plaque removal (PMPR) procedure performed by a licensed clinician. Ultrasonic debridement was carried out using the Piezon^®^ Prophylaxis Master with Piezon^®^ PS instrument (EMS, Nyon, Switzerland), followed by full-mouth air polishing using the AIRFLOW^®^ Prophylaxis Master^®^ handpiece with Airflow^®^ PLUS powder (EMS, Nyon, Switzerland). The procedure ensured the complete removal of supragingival plaque and biofilm. A 24-hour washout period was observed before initiating the assigned mouthrinse. This standardized intervention allowed for a “zero baseline” condition, ensuring that subsequent plaque accumulation reflected the efficacy of the test and control mouthrinses.

Participants were then randomly assigned to one of four groups using a computer-generated randomization list. The groups were defined by the mouthwash intervention:

***Group 1 (propolis)***: mouthwash containing a standardized propolis extract solution (10% propolis, alcohol-free).

***Group 2 (green tea)****:* mouthwash containing green tea extract (5% Camellia sinensis extract, alcohol-free).

***Group 3 (chlorhexidine, positive control)****:* 0.2% chlorhexidine gluconate mouthwash (standard antiseptic control).

***Group 4 (placebo, negative control)****:* placebo mouthwash (distilled water with mint flavor, no active ingredient). 

The propolis mouthrinse used in this study contained a 10% concentration of a commercially available, standardized, alcohol-free propolis extract. The formulation was aqueous, using emulsification and surfactant-assisted dispersion techniques to solubilize the resinous components of propolis and ensure the stability and bioavailability of its active compounds.

All mouthwashes were similar in appearance (light amber-colored) and were dispensed in identical bottles coded by group to maintain blinding of the examiner. All mouthwash formulations used in this study were alcohol-free. The participants were blinded to whether they had a natural or control rinse; however, because chlorhexidine has a distinct taste, complete blinding of the participants was not possible. The clinical examiner conducting the plaque assessments was blinded to the group allocation.

### 2.3. Intervention and OHI Protocol

**Figure 2:**
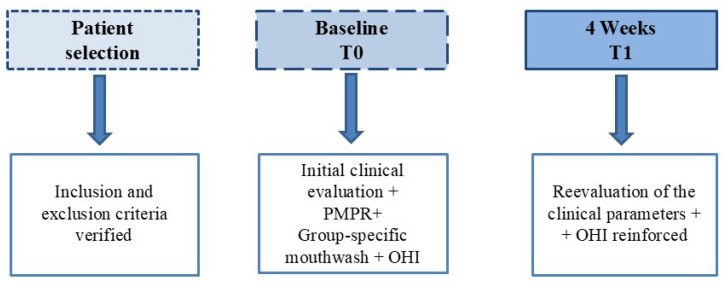
Time diagram.

All participants were instructed on how to use the mouthrinse and maintain their usual oral hygiene: they were to rinse with 15 mL of the assigned solution for 30 s twice daily (morning and night) after tooth brushing and refrain from eating or drinking for 30 min after rinsing. They continued their regular tooth brushing (twice daily using a fluoride toothpaste) and flossing habits throughout the 4-week trial but were asked to avoid using any other mouthwash or oral product aside from those provided. Compliance was monitored via a daily log and by weighing the returned mouthwash bottles to estimate usage (Figure 2).

The primary outcome was dental plaque accumulation, measured by the plaque index (PI), as described by Silness and Löe (1964) [18]. For each participant, the PI was assessed at baseline (before PMPR) and at the end of the 4-week intervention. The plaque index was scored as a full-mouth index on all teeth (six per tooth), with an individual score from 0 to 3. The examiner was a calibrated dentist who performed all plaque scoring under identical conditions at baseline and follow-up.

The secondary outcomes included any reported adverse effects (such as oral irritation, burning sensation, changes in taste, or tooth staining observed) and participant compliance. The primary focus of the study was plaque; however, we also documented the gingival index (GI), as per Löe and Silness (1963) [19], at baseline and after 4 weeks to assess the gingival inflammation, ensuring that short-term alterations in plaque were not correlated with unforeseen gingival consequences. Additional baseline clinical data, including bleeding on probing (BoP) and probing depth (PD), were documented for all groups using a periodontal probe (UNC-15, Hu-Friedy, Chicago, IL, USA). The probing depth (PD) was assessed on six dental surfaces: distobuccal, mid-buccal, mesiobuccal, distolingual, mid-lingual, and mesiolingual, with the average probing depth value calculated. The BoP was documented during the PD assessment by evaluating the occurrence of bleeding lasting up to 30 s. The periodontal condition of the participants was classified as gingivitis (BoP ≥ 10%; PPD < 3 mm; absence of interdental CAL) or periodontitis (BoP ≥ 10%; PPD > 3 mm; presence of interdental CAL in at least two non-adjacent teeth).

The clinical measures were evaluated at six locations per tooth in each study participant. The probing depth (PD) was assessed to the nearest whole millimeter from the gingival edge to the deepest penetration of the probe tip into the apical gingival tissue, utilizing a UNC-15 periodontal probe, with the probe placed parallel to the tooth’s long axis. Prior to the study, the examiner (specialist of periodontology) was calibrated. The intra-examiner calibration for reliability testing resulted in κ = 0.92 for repeated measurements of PD in two quadrants of five patients other than the patients recruited for the study (to complete the evaluations needed for this study in a reliable and accurate manner that was consistent with current standards for clinical periodontal studies).

### 2.4. Statistical Analysis

Data were analyzed using MedCalc 23.0.6 for Windows (© 1993–2024 MedCalc Software Ltd., Ostend, Belgium). The values for each group were summarized as a mean ± standard deviation (SD). The primary analysis compared post-intervention plaque indices among the four groups using a one-way analysis of variance (ANOVA). Before the ANOVA, the normality of the data and the homogeneity of the variances were confirmed (Shapiro–Wilk test and Levene’s test, respectively). For pairwise comparisons between the groups, Tukey’s honest significant difference post hoc test was applied. A paired *t*-test was used to compare within-group changes in PI from baseline to 4 weeks. The significance level was set at *p* < 0.05 for all tests.

### 2.5. Sample Size Calculation

The sample size was determined using G*Power version 3.1.9.7 (Heinrich Heine University Düsseldorf, Düsseldorf, Germany) based on a one-way ANOVA model designed to detect differences in plaque index (PI) reductions among four independent groups (propolis, green tea, chlorhexidine, and placebo). A moderate-to-large effect size (f = 0.40) was assumed, with a significance level (α) of 0.05 and a statistical power (1–β) of 0.80, based on data from a previous randomized clinical trial evaluating the antiplaque efficacy of natural mouthrinses compared to chlorhexidine [20]. The minimum required sample size was calculated to be 52 participants (approximately 13 per group). To account for potential dropouts or non-compliance, the final sample size was increased to 60 participants, with 15 individuals randomly assigned to each group. This sample size was considered appropriate given the pilot nature of the trial and its objective of evaluating preliminary efficacy and feasibility.

## 3. Results

### 3.1. Participant Flow and Baseline Characteristics

**Figure 3:**
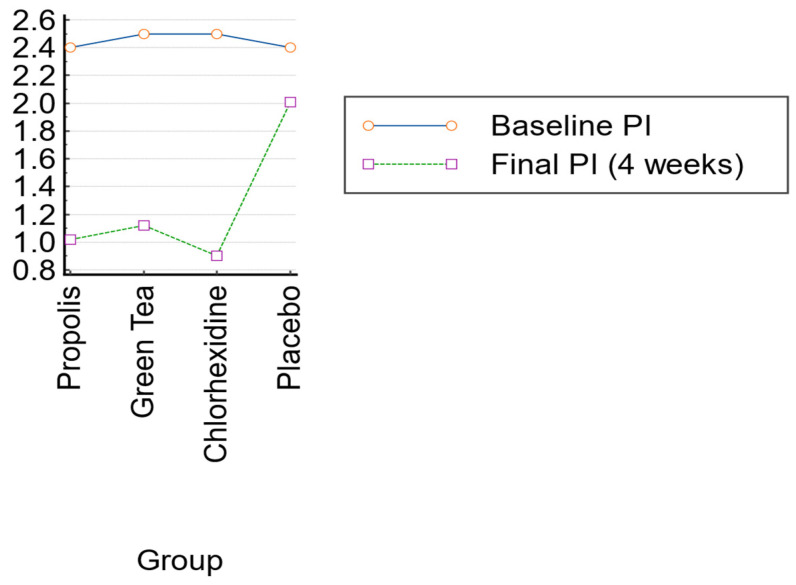
Plaque reduction between groups at baseline and at 4 weeks.

**Figure 4:**
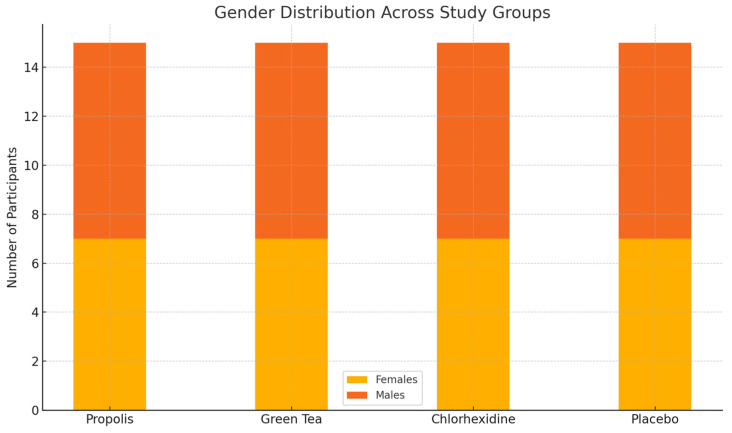
Gender distribution across the study groups.

Sixty participants were enrolled and randomized equally into four groups (n = 15 each): propolis, green tea, chlorhexidine, and placebo. All participants completed the 4-week study, and no dropouts occurred. The groups were comparable in terms of baseline characteristics. The study population consisted of 60 adults (28 females and 32 males), aged between 18 and 50 years (mean age = 34.2 ± 8.6 years), with similar distribution across the four study groups (Figure 3 and Figure 4).

**Figure 5:**
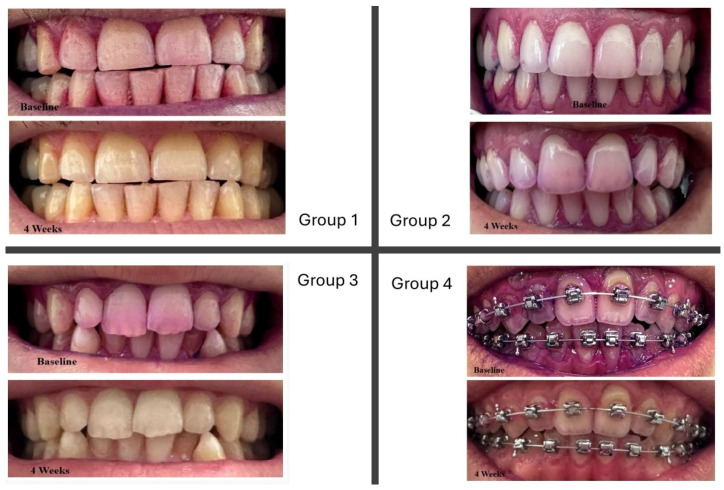
Representative intraoral images showing plaque disclosure at baseline (**top row**) and after 4 weeks (**bottom row**) for each study group. Plaque accumulation was visualized using a disclosing agent, highlighting differences in oral hygiene effectiveness following the use of various mouthrinses.

The mean baseline plaque index (PI) prior to professional mechanical plaque removal (PMPR) was as follows: propolis: 2.4 ± 0.3; green tea: 2.5 ± 0.4; chlorhexidine: 2.5 ± 0.3; placebo: 2.4 ± 0.3. There were no statistically significant differences in the baseline plaque scores between the groups (*p* = 0.88). The baseline gingival index (GI) scores were also similar (mean GI ≈ 1.2, indicative of mild gingivitis; *p* > 0.9). All participants complied with the pre-trial instructions to abstain from other mouthrinses and had no recent antibiotic use, in accordance with the exclusion criteria (Figure 5).

### 3.2. Plaque Index After 4 Weeks

**Figure 6:**
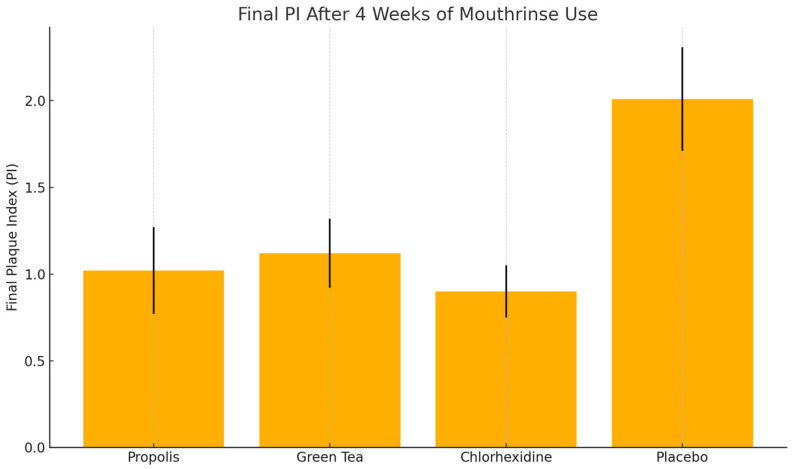
Plaque reduction between the groups at 4 weeks, the vertical lines represent the standard deviation (SD), indicating the variability of PI scores within each group.

After 4 weeks of twice-daily rinsing, the plaque index scores decreased in all groups, with the most significant reductions observed in the propolis, green tea, and chlorhexidine groups. The propolis group resulted in PI = 1.02 ± 0.25, with a ~58% reduction from baseline. The green tea group resulted in PI = 1.12 ± 0.20, with a ~55% reduction from baseline. The chlorhexidine group resulted in PI = 0.90 ± 0.15, with a ~64% reduction from baseline. The placebo group resulted in PI = 2.01 ± 0.30, with a ~16% reduction; plaque re-accumulated close to baseline levels. A one-way ANOVA revealed a highly significant difference in the final plaque scores between the groups (*p* < 0.0001). The post hoc comparisons showed that the propolis and green tea groups both had significantly lower PI scores compared to the placebo (*p* < 0.001) (Figure 6).

The propolis group’s score was statistically equivalent to the chlorhexidine group (*p* = 0.40), indicating similar efficacy. The green tea group had a slightly higher PI than the chlorhexidine group (*p* = 0.03), although the difference was small. There was no significant difference between the propolis and green tea groups (*p* = 0.60). The within-group analyses confirmed statistically significant reductions in plaque indices from baseline for the propolis, green tea, and chlorhexidine groups (paired *t*-tests, *p* < 0.001 for each). The placebo group’s slight reduction was not statistically significant (*p* = 0.08).

By the end of the trial, the percentage of participants achieving “good plaque control” (PI ≤ 1.0) was as follows: propolis: 58%; green tea: 55%; chlorhexidine: 57%; placebo: 16% (Table 1).

### 3.3. Adverse Effects

No serious adverse events were reported. Mild, transient effects were noted for two participants (13%) in the propolis group, who reported a slight burning sensation during the first few days of use, which was resolved spontaneously. One participant (7%) in the green tea group reported a mild bitter aftertaste. No participants reported tooth staining, likely due to the short study duration (chlorhexidine-related staining typically occurs with prolonged use).

### 3.4. Gingival Index Changes

The gingival index scores improved slightly and had a similar trend in all groups by 4 weeks (average GI reduction of ~0.2 in each group, including the placebo), consistent with the general improvement in oral hygiene after baseline PMPR; there were no significant differences in the gingival index change between the groups. This suggests that none of the mouthwashes caused irritation or worsened the gingival condition.

### 3.5. Compliance

Compliance was high across all groups, based on self-reports and returned bottle volumes, and approximately 93.33% of all instructed rinses were used across the participants. All participants reported using the rinse at least once per day on average, and 86.66% reported a twice-daily use as instructed. There was no significant difference in the compliance between the groups. Thus, the efficacy differences observed can be attributed to the mouthwash contents rather than the disparities in the usage.

### 3.6. BoP After 4 Weeks

Table 2 shows the BoP reduction between the groups. The propolis and chlorhexidine (CHX) groups had the greatest reductions in bleeding on probing (BoP), with 36.55% and 35.89% reductions, respectively, indicating strong effects. Green tea reduced BoP by 23.06%, and the placebo group showed the smallest improvement, with 12.07%. Propolis showed similar effectiveness as chlorhexidine in reducing gingival bleeding and can be considered a natural alternative for improving oral health. Green tea offers moderate benefits, whereas the placebo has little effect.

## 4. Discussion

The present pilot trial investigated the short-term antiplaque efficacy of two natural mouthrinses—10% propolis and 5% green tea—compared to 0.2% chlorhexidine (CHX) and a placebo. Both natural agents demonstrated significant plaque reduction, with propolis showing efficacy comparable to CHX and green tea showing slightly lower, yet still clinically relevant, results. These findings support the growing evidence that specific natural compounds can be effective adjuncts to mechanical plaque control and, in some cases, potential alternatives to CHX.

Chlorhexidine gluconate remains the gold standard in chemical plaque control due to its substantivity and broad-spectrum antimicrobial action against both aerobic and anaerobic oral pathogens [1,6]. Its cationic nature allows it to bind to negatively charged oral surfaces and bacterial membranes, causing disruption of the cell wall and leakage of intracellular components [21,22,23]. However, as widely reported, the prolonged use of CHX is associated with undesirable side effects, including dental staining, altered taste, and mucosal irritation [4,24].

Our results confirm the well-documented plaque-inhibitory properties of CHX, with the greatest reduction in plaque index (64%) observed in this group. These findings are consistent with prior trials demonstrating CHX’s efficacy over periods of 2–6 weeks [6]. Notably, propolis achieved a 58% reduction in the plaque index, which was statistically equivalent to CHX. This aligns with the results of Mathur et al. (2018), [25] who found no significant difference between propolis and CHX in a systematic review of randomized controlled trials [25]. Other studies have attributed this effect to the high content of flavonoids and phenolic acids in propolis, which interfere with bacterial adhesion, glucosyltransferase activity, and biofilm matrix integrity [26,27].

Green tea extract, which led to a 55% plaque reduction, also demonstrated significant effectiveness compared to the placebo, although it was slightly inferior to CHX (*p* = 0.03). This mirrors the findings of Steinberg et al. (2020) and Singh et al. (2020), where green tea and neem-based rinses achieved meaningful antiplaque outcomes, though often marginally lower than CHX [6,7]. The catechins in green tea—especially EGCG—have been shown to inhibit *Streptococcus mutans* acidogenesis, suppress bacterial adherence, and reduce glucan formation [28,29,30,31].

The clinical implications of these results are particularly relevant in light of the need for long-term, well-tolerated plaque control strategies. While CHX is typically recommended for short-term use (e.g., postsurgical or during acute gingival inflammation), natural agents such as propolis and green tea may offer a safer alternative for longer-term maintenance. In our study, only mild, transient side effects were observed in the natural rinse groups, and no participants reported staining, which is a frequent drawback of CHX reported in the literature [24,32].

A notable strength of our study lies in the standardization of initial plaque conditions through professional mechanical plaque removal (PMPR) before intervention. This allowed for the accurate assessment of new plaque accumulation over the 4-week period, isolating the effects of the tested mouthrinses. Our findings also resonate with a broader shift toward natural, plant-based therapies in preventive dentistry, supported by increasing patient demand for chemical-free and sustainable oral health products [33].

However, the causative relationship between bioactive compounds and clinical efficacy is complex. Factors such as concentration, bioavailability, formulation stability, and the interaction of active constituents with the oral microbiome all play a role in determining effectiveness. For instance, the slightly inferior result of green tea compared to CHX may reflect variability in EGCG stability and delivery, which has been noted in previous in vitro studies [31]. In contrast, propolis formulations appear to provide a more stable and concentrated delivery of active agents.

Beyond plaque control, recent studies have also explored adjunctive antimicrobial agents for periodontal and peri-implant diseases. Recent studies (2023–2024) demonstrated significant improvements in clinical outcomes using locally delivered antibiotic gels, such as doxycycline or piperacillin-tazobactam, as adjuncts to mechanical debridement [17,21,22]. These findings support the ongoing investigation into bioactive topical therapies, of which propolis may be a viable candidate.

Despite its strengths, our study has limitations. The trial was limited to healthy adults over a short 4-week duration, which may not fully capture the long-term performance or potential side effects of the rinses. Furthermore, the single-blind design—although necessary due to taste differences—may have introduced minor behavioral bias. Lastly, no microbiological analyses were performed; future studies should explore how these natural agents modulate the oral microbiota, including their potential to selectively inhibit pathogenic species while preserving the commensal flora.

In conclusion, this trial adds to the growing body of evidence suggesting that natural mouthrinses, particularly those containing propolis, offer promising antiplaque efficacy comparable to chlorhexidine, with fewer side effects. Further long-term, multicenter studies are needed to validate these findings and define their role in clinical practice.

It is important to consider the potential influence of the Hawthorne effect, whereby participants may modify their behavior due to the awareness of being observed within a clinical research context. In the present trial, all participants received professional mechanical plaque removal (PMPR) at baseline and were provided with standardized oral hygiene instructions. These interventions, coupled with the structured nature of study participation, likely enhanced motivation and adherence to personal oral hygiene practices. This behavioral response may account, at least in part, for the modest reduction in plaque scores observed in the placebo group. Such effects highlight the necessity of accounting for participant reactivity when interpreting short-term clinical outcomes in oral health studies.

Despite the promising results, this pilot trial has several limitations and did not include a microbiological analysis of dental plaque. Due to limited scope and resources, bacterial cultures and molecular profiling were not performed, preventing us from directly assessing changes in the oral microbiota. Additionally, the 4-week intervention period provides only a short-term perspective on efficacy and does not allow for conclusions regarding long-term outcomes or cumulative benefits. The study sample consisted exclusively of healthy young adults, which may limit the generalizability of the findings to other populations, such as older individuals or those with systemic or advanced oral health conditions. Furthermore, the single-blind design—necessary due to the distinguishable taste of the active mouthrinses—may have introduced minor behavioral bias, despite the consistent oral hygiene instructions and monitoring across all groups.

## 5. Conclusions

In conclusion, this study provides evidence that certain natural compounds can effectively reduce bacterial plaque levels on teeth. The use of a 10% propolis-based mouthwash resulted in a plaque reduction comparable to that achieved by a standard 0.2% chlorhexidine rinse, while the 5% green tea extract mouthwash also significantly inhibited plaque accumulation compared to placebo. These findings confirm the study’s objective by demonstrating that natural substance-based interventions can serve as effective antiplaque agents. The research hypothesis was supported, particularly in the case of propolis, which matched the efficacy of the conventional antiseptic.

## Figures and Tables

**Table 1:** Plaque reduction between the groups at baseline and 4 weeks.

Group	Baseline PI (Mean ± SD)	Final PI (Mean ± SD)	PI Reduction (%)	Good PI Control (% PI ≤ 1.0)	Mild Side Effects (%)
**Propolis**	2.4 ± 0.3	1.02 ± 0.25	58	58	13% (burning)
**Green Tea**	2.5 ± 0.4	1.12 ± 0.20	55	55	7% (bitter taste)
**CHX**	2.5 ± 0.3	0.90 ± 0.15	64	57	0%
**Placebo**	2.4 ± 0.3	2.01 ± 0.30	16	16	0%

PI, plaque index; CHX, chlorhexidine; SD, standard deviation.

**Table 2:** BoP reduction between the groups at baseline and 4 weeks.

Group	Baseline BoP (Mean ± SD)	Final BoP (Mean ± SD)	BoP Reduction (%)
Propolis	35.05 ± 11.22	22.24 ± 13.15	36.55%
Green Tea	28.14 ± 23.14	21.65 ± 13.23	23.06%
CHX	28.42 ± 17.78	18.22 ± 22.47	35.89%
Placebo	31.98 ± 19.34	28.12 ± 30.29	12.07%

## Data Availability

The data presented in this study are available on request from the corresponding author. The data are not publicly available due to privacy and ethical reasons.

## Abbreviations

The following abbreviations are used in this manuscript:

PMPR	professional mechanical plaque removal
PI	plaque index
SD	standard deviation
n	number
GI	gingival index
